# The relation of secondary sex ratio and miscarriage history with *Toxoplasma gondii* infection

**DOI:** 10.1186/s12879-018-3228-0

**Published:** 2018-07-05

**Authors:** Saeedeh Shojaee, Aref Teimouri, Hossein Keshavarz, Sanaz Jafarpour Azami, Sahar Nouri

**Affiliations:** 1 Department of Medical Parasitology and Mycology, School of Public Health, Tehran University of Medical Sciences, P.O. Box: 1417613191, Pour Sina Street, Ghods Avenue, Enghelab Avenue, Tehran, Iran; 2 Students Scientific Research Center, Tehran University of Medical Sciences, Tehran, Iran; 3 Department of Epidemiology and Biostatistics, School of Public Health, Tehran University of Medical Sciences, Tehran, Iran

**Keywords:** *Toxoplasma gondii*, Secondary sex ratio, Miscarriage, Cord blood serum, ELISA

## Abstract

**Background:**

*Toxoplasma gondii* is a protozoan parasite with worldwide distribution, infecting a broad-range of humans and warm-blooded animals. In the current study, role of this parasite on secondary sex ratio and risk of miscarriage was investigated.

**Methods:**

In this cross-sectional study, 850 cord blood samples were collected in Tehran, Iran, 2014–2015. Enzyme-linked immunosorbent assay (ELISA) was used to assess anti-*Toxoplasma* IgG in samples. Information such as sex of the neonates and age, number of previous pregnancies and history of miscarriage of the mothers were recorded in questionnaires. Logistic regression analysis was used to assess the possible relationship between the latent toxoplasmosis and the highlighted parameters.

**Results:**

Logistic regression analysis showed that the odds of having a male neonate in seropositive women is nearly 64% higher than that in seronegative women (OR = 1.64, CI_95_ = 1.16–2.33, *P* = 0.005). The odds ratio of having male neonate increased to 2.10 (CI_95_ = 1.24–3.57, *P* = 0.006) in high-titer seropositive women, compared to that in seronegative control group. The odds of having a miscarriage history was approximately two and a half times greater in seropositive women than in seronegative ones (OR = 2.45, CI_95_ = 1.56–3.87, *P* < 0.001). The odds ratio of having miscarriage increased to 2.76 (CI_95_ = 1.61–4.73, *P* < < .001) in low-titer seropositive women, compared to that in seronegative control group.

**Conclusion:**

Results of the current study have shown that *T. gondii* infection affects secondary sex ratio in human offspring and can be addressed as one of the major miscarriage causes in women.

## Background

Toxoplasmosis is one of the most prevalent parasitic diseases, caused by a coccidian protozoan, *Toxoplasma gondii* [1]. Prevalence of *T. gondii* infection varies 10–80% in various regions of the world. Large differences have also been reported in Iran, with a prevalence of 39.8% in Golestan Province and 28.8% in Kerman Province [2, 3]. The main routes of human infection include consumption of raw or undercooked meat containing tissue cysts and ingestion of oocysts via contaminated waters or vegetables [4]. Vertical transmission of rapidly dividing tachyzoites from the pregnant mother to developing fetus is the other route of human infection. This infection route could lead to abortion, chorioretinitis or serious developmental disorders such as hydrocephaly and microcephaly [5]. Therefore, the accurate diagnosis of acute maternal toxoplasmosis in pregnant women is critical [6]. The acquired *T. gondii* infection in immunocompetent hosts is usually benign and results in latent infections with formation of cysts in brain, lungs and other tissues [7]. Latent toxoplasmosis is clinically asymptomatic but may be complicated by the increased risk of psychiatric and neurological abnormalities and personality changes such as bipolar disorder, obsessive-compulsive disorder, recurrent migraine, cryptic epilepsy, autism, suicide attempt, homicide and brain tumor [8].

The secondary sex ratio (the ratio of males to females at birth) is nearly 0.51 in human [9], which is affected by factors including age of parents [10], stress, immunosuppression, paternal endocrine disruption such as diabetes [11–13] and socioeconomic status of the parents [14]. Until now, three studies have assessed the effects of latent *Toxoplasma* infection on sex ratio at birth in humans and mice and all three found significant effects [15–17]. In contrast, effects of latent toxoplasmosis on risk of miscarriage are still being discussed by researchers [18–20]. Therefore, the present study was carried out to assess possible effects of *T. gondii* infection on secondary sex ratio and risk of miscarriage in 850 cord blood serum samples from delivered women in Tehran, Iran, 2014–2015.

## Methods

### Patients

In this cross-sectional study, 850 cord blood samples were collected in Tehran, Iran, 2014–2015. Sera were separated from the whole blood samples and stored at − 20 °C until use. Information such as sex of the neonates and age, number of previous pregnancies and history of miscarriage of the women were recorded in questionnaires and used for statistical analysis.

### Serological tests

Enzyme-linked immunosorbent assay (ELISA) was used to assess anti-*Toxoplasma* IgG in cord blood serum samples. The cut off value of optical density (OD) was calculated based on a protocol by Hillyer et al. [21]. Optical density of each sample was compared to the cut off value and recorded as positive or negative result.

### Preparation of soluble antigens of *T. gondii*

Antigens were prepared as previously described [22]. Briefly, tachyzoites of *T. gondii*, RH strain, were inoculated into peritoneum cavity of BALB/c mice. After three days, the tachyzoites were harvested by peritoneum wash, then washed for three times, sonicated and centrifuged at 14000 g for 1 h at 4 °C. Soluble antigens were collected and protein densities were assessed using Bradford method.

### Enzyme-linked immunosorbent assay (ELISA)

In general, 96-well microplates (Nunk, Germany) were coated with 5 μg/ml of the soluble antigen of *T. gondii*, RH strain, and stored at 4 °C overnight. Cord blood serum samples were diluted 1:200 in PBST (phosphate buffered saline, tween 20) and added to each well of the microplates. After 1 h of incubation and three times of wash, 100 μl of anti-human IgG conjugated with horseradish peroxidase (HRP) (Dako, Denmark) were added to the wells in dilutions of 1:500 in PBST. After incubation and wash steps, substrate of ortho-phenylenediamine (OPD) (Sigma-Aldrich, USA) was added to the wells. Reactions were stopped by adding a 20% sulfuric acid solution and the OD was recorded using an automated ELISA reader (Biotek, USA) at 490 nm [23].

### Statistical analysis

Statistical analysis was carried out using SPSS Software v.21.0 (IBM Analytics, USA) [24]. Association between the sex of newborns (binary) or miscarriage history (binary) as dependent variables and two independent variables including *Toxoplasma*-seropositivity (binary) and age of the mothers (continuous) was assessed using logistic regression model. Furthermore, multiple logistic regression analysis was used to control the effect of potential confounding factors on the sex of neonates and history of miscarriage. Data description was carried out by calculating frequencies and 95% confidence intervals. Differences were considered as significant when *P* ≤ 0.05.

## Results

**Fig. 1:**
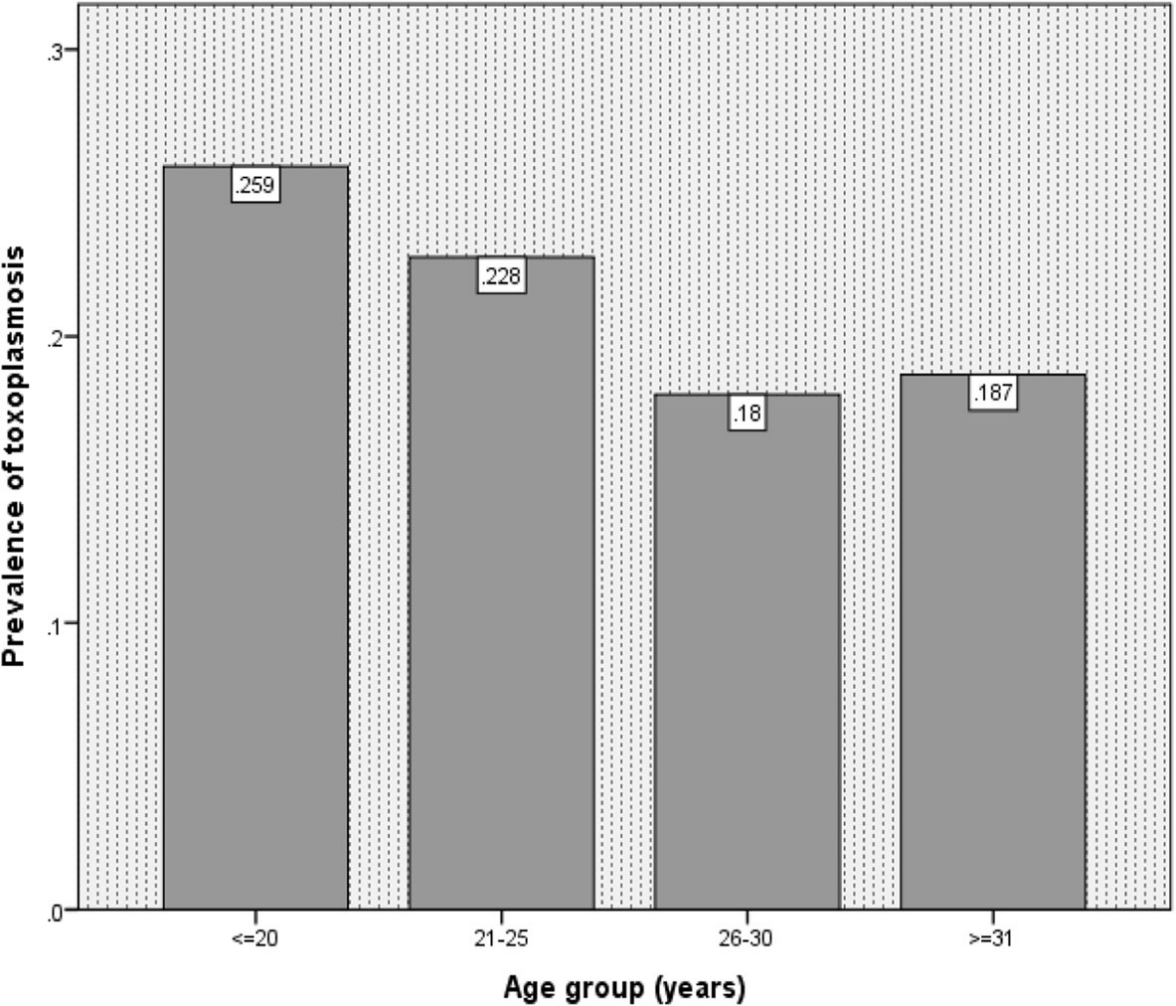
Prevalence of anti-*Toxoplasma* IgG in 850 cord blood serum samples in particular age groups of participants using ELISA

Of 850 cord blood serum samples, 166 samples were positive for anti-*Toxoplasma* IgG; therefore, the overall anti-*Toxoplasma* antibody prevalence was 19.5% in this study. The average age of the participants was approximately 28.7 years with 95% confidence interval (28.4–29.1). Participants were divided into four age groups of ≤20, 21–25, 26–30 and ≥ 31. The majority of the pregnancies (334/850, 39.3%) occurred between 26 and 30 years of age. According to the age of women, the prevalence of anti-*Toxoplasma* IgG in 850 cord blood serum samples of participants was as follows: ≤ 20 age group, 7/27 (25.9%); early 20s, 43/189 (22.8%); late 20s, 60/334 (18%); and ≥ 31 age group, 56/300 (18.7%) (Fig. 1).

**Fig. 2:**
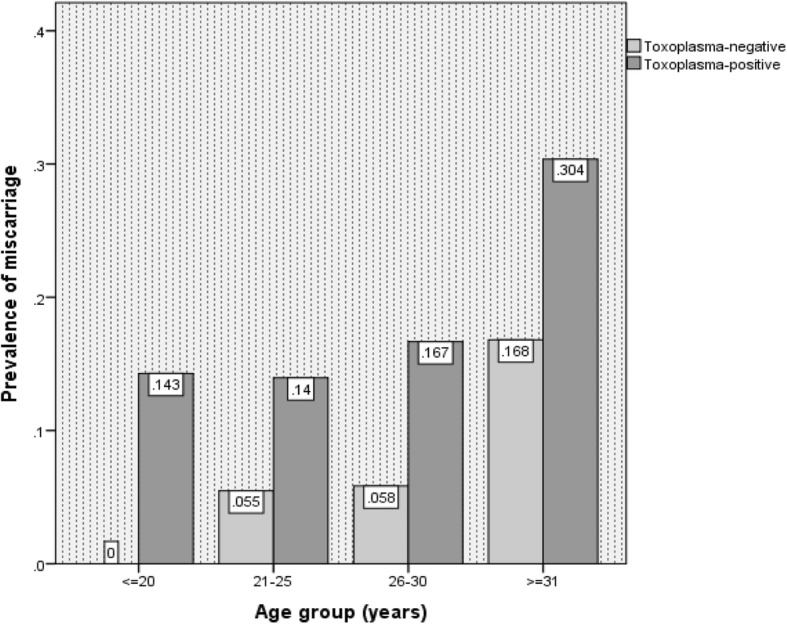
Prevalence of miscarriage in particular age groups of 166 *Toxoplasma*-positive and 684 *Toxoplasma*-negative subjects using ELISA

The highest frequency of miscarriage in *Toxoplasma*-positive and *Toxoplasma*-negative subjects was observed in ≥31 age group (Fig. 2), however, the association between the prevalence of toxoplasmosis and age of the women was not statistically significant (*P* = 0.4). Furthermore, no significant associations were seen between the miscarriage history and age of the women in seropositive participants (*P* = 0.15). Of 850 studied pregnant women, 444 delivered male neonates and 99 had a history of miscarriage.

### Association of anti-*Toxoplasma* IgG with sex ratio

The *Toxoplasma*-seropositivity was significantly associated with having a male neonate (OR = 1.64, CI_95_ = 1.16–2.33, *P* = 0.005); 103 out of 166 (62.1%) newborns from seropositive mothers were males while 341 out of 684 (49.9%) newborns from seronegative mothers were males. The OR of having male neonates increased up to 2.10 (CI_95_ = 1.24–3.57, *P* = 0.006) in seropositive women with a high concentration of anti-*Toxoplasma* antibodies (OD > 0.75), compared to that in *Toxoplasma*-negative group. No significant differences were seen in OR of having male neonates in low-titer group (OD ≤ 0.75), compared to that in negative control group (OR = 1.4, CI_95_ = 0.91–2.16, *P* = 0.12).

Results from the multiple logistic regression analysis showed that the *Toxoplasma*-seropositivity was significantly associated with having a male neonate (OR = 1.66, CI_95_ = 1.16–2.35, *P* = 0.005) after controlling for effects of miscarriage history in the model. Furthermore, the OR of having male neonates in high-titer and low-titer groups of seropositive women was 2.12 (CI_95_ = 1.25–3.6, *P* = 0.006) and 1.4 (CI_95_ = 0.91–2.16, *P* = 0.12) respectively, compared to that in toxoplasma-seronegative women. Association between the age of women and sex of the neonates was not statistically significant (OR = 0.98, CI_95_ = 0.9–1, *P* = 0.19).

### Association between anti-*Toxoplasma* IgG and miscarriage

In 99 women with miscarriage history (mean age 30.87 years, CI_95_ = 29.9–31.82), anti-*Toxoplasma* IgG antibodies were found in 34.3% of cases while in 751 women without miscarriage history (mean age 28.42 years, CI_95_ = 28.15–28.81) the anti-*Toxoplasma* IgG antibodies were found in 17.6% of cases. Results of the logistic regression showed that *Toxoplasma*-seropositivity was statistically associated to miscarriage (OR = 2.45, CI_95_ = 1.56–3.87, *P* < 0.001). The OR of having miscarriage increased up to 2.76 (CI_95_ = 1.61–4.73, P < 0.001) in seropositive women with a low concentration of anti-*Toxoplasma* antibodies (OD ≤ 0.75), compared to that in *Toxoplasma*-negative group. The OR of having miscarriage in high-titer group (OD > 0.75) was 2.04 (CI_95_ = 1.04–4.00, *P* = 0.038). According to the results from logistic regression, there was a direct association between miscarriage history and age of mothers. The OR of miscarriage increased by 1.94 (CI_95_ = 1.45–2.61) for every five years increase in age of mothers.

Results from the multiple logistic regression analysis showed that the association between *Toxoplasma*-seropositivity and having miscarriage history was statistically significant (OR = 2.63, CI_95_ = 1.64–4.19, *P* < 0.0001) after controlling for effects of the age of mothers in the model. Moreover, the OR of having miscarriage history in high-titer and low-titer groups of *Toxoplasma*-seropositive women was 2.06 (CI_95_ = 1.04–4.1, *P* = 0.04) and 3.24 (CI_95_ = 1.85–5.68, P < 0.0001) respectively, compared to that in seronegative women. No significant association was found between the concentration of anti-*Toxoplasma* antibodies and the miscarriage history in seropositive women (OR = 0.74, CI_95_ = 0.34–1.6, *P* = 0.45). In addition, association between the history of miscarriage and sex of the neonates were not statistically significant (OR = 1.01, CI_95_ = 0.67–1.54, *P* = 0.9).

Association of latent toxoplasmosis with maternal age at pregnancy was assessed using analysis of variance in a subset of primiparous women. Totally, 412 (48.4%) primiparous women were included in the current study. The estimated mean age for IgG-positive and IgG-negative primiparous women with no history of miscarriage included 25.93 (CI_95_ = 25.04–26.82) and 26.4 (CI_95_ = 25.98–26.83), respectively. Analysis of variance showed no significant differences between the two mean values (*P* = 0.58).

## Discussion

The dormant stage of *T. gondii* infection in humans is characterized by the presence of tissue cysts in brain and other organs. From the clinical point of view, these life-long tissue cysts are almost asymptomatic in infected individuals [8]. In recent years, effects of asymptomatic latent toxoplasmosis in human life have been investigated [8]. Possible relationships of latent toxoplasmosis with mental disorders such as schizophrenia [25], Alzheimer^’^s disease [26] and cognitive disorder [27] have been studied in humans and animal models. Increased reaction times [28] in *Toxoplasma*-infected individuals may explain the high-rate traffic accidents by these patients [29]. *Toxoplasma gondii* can affect personality of the infected patients [8]. The *T. gondii* infected men are nearly 3 cm taller than *T. gondii* free men, having further muscles and dominant faces. These differences may be associated with different testosterone levels in *T. gondii* positive and *T. gondii* negative men [30, 31]. Effects of *T. gondii* infection on endocrine and immune systems have been demonstrated repeatedly and their association with secondary sex ratio is not surprising [32]. According to results of the study by Flegr et al., latent toxoplasmosis has surprisingly serious effects on public health. Furthermore, *T. gondii* is one of the most important factors affecting variation of offspring sex ratio over the world [33].

Results of the present study have shown that *T. gondii* infection is significantly associated with having male neonates and that the association is statistically significant. The OR of having male neonates in *T. gondii* seropositive women was approximately 64% higher than that in seronegative women. A retrospective cohort study in Czech Republic (1996–2004) has shown that of 1803 neonates, the secondary sex ratio increased in 454 *Toxoplasma*-positive women (percentage of males = 0.608), compared to 1349 *Toxoplasma*-negative women (percentage of males = 0.527; *P* = 0.0027) [16]. In the current study, the OR of having male neonates increased in women with high-titer anti-*Toxoplasma* IgG as the value of 1.64 in seropositive women reached 2.10 in women with high-titer antibodies. A similar study by Kankova et al. (2007) showed that women with high concentration of anti-*Toxoplasma* IgG gave birth to 250 male neonates per 100 female neonates, while women with low concentration of anti-*Toxoplasma* of IgG gave birth more female than male neonates [16]. In experimental models, mice with *T. gondii* infection had a greater sex ratio, compared to control group [17]. Increased sex ratio could be explained by the effects of latent toxoplasmosis on immunosuppression mechanisms in mice and humans [34], as the secondary sex ratio is affected by significant modulation of immune responses and cytokine production in infected mice. *Toxoplasma* parasite could rescue male fetuses with developmental disorders by inducing immunosuppression mechanisms [35].

Seroprevalence of toxoplasmosis in women with abortion events has been recorded high (17–43.8%) in African and Asian countries [36–38]. In the present study, relatively high seroprevalence (34.3%) of *T. gondii* infection was detected in cord blood serum samples of women with miscarriage history and the relationship between these two parameters was statistically significant. The odds of having miscarriage was approximately two and a half times higher for the seropositive women than for the seronegative women. The OR of having miscarriage increased to the value of 2.76 in low-titer seropositive women, compared to that in seronegative control group. The OR of having miscarriage in high-titer group was only 2.04. Moreover, the association between presence of anti-*Toxoplasma* IgG and miscarriage history in seropositive women was not statistically significant. Similar findings were reported by Sharf et al., 1973; Mahajan et al., 1976; Sahwi et al., 1995 and Al-Hamdani, 1997 [39–42]. In a study by Alvarado-Esquivel et al. (2016) in Mexico, association of *Toxoplasma* infection and abortion in pregnant women of rural areas with high seropositivity rate was reported [43]. In 2011, Pavlinova et al. recorded 42.1% anti-*Toxoplasma* IgG seropositivity in 530 women with recurrent miscarriage which were significantly (*P* < 0.0004) greater than that in control groups (25.1%) [44]. In a study by Turbadkar et al., bad obstetric history (BOH) was recorded in 42.1% of *Toxoplasma* seropositive women [45]. In the present study, history of miscarriage in pregnant women and secondary sex ratio of offspring were statistically linked to *T. gondii* infection.

## Conclusion

In conclusion, *T. gondii* infection affects secondary sex ratio in humans and can be addressed as one of the major miscarriage reasons in women.

## Abbreviations

BOH: Bad obstetric history
ELISA: Enzyme-linked immunosorbent assay
HRP: Horseradish peroxidase
IBM: International business machines corporation
IgG: Immunoglobulin G
OD: Optical density
OPD: Ortho phenylenediamine
OR: Odds ratio
PBST: Phosphate buffered saline, tween 20
SPSS: Statistical package for the social sciences
